# Ethnoveterinary treatments by dromedary camel herders in the Suleiman Mountainous Region in Pakistan: an observation and questionnaire study

**DOI:** 10.1186/1746-4269-6-16

**Published:** 2010-06-21

**Authors:** Abdul Raziq, Kerstin de Verdier, Muhammad Younas

**Affiliations:** 1 President of Society of Animal, Veterinary and Environmental Scientists (SAVES), Pakistan; 2 Department of Animal health and Antibiotic Strategies, National Veterinary Institute, 761 89 Uppsala, Sweden; 3 Department of Livestock Management, Faculty of Animal Husbandry, University of Faisalabad, Pakistan

## Abstract

**Background:**

The Suleiman mountainous region is an important cradle of animal domestication and the habitat of many indigenous livestock breeds. The dromedary camel is a highly appreciated and valued animal and represents an important genetic resource. Camel herders, living in remote areas, have developed their own ways to treat diseases in camels, based on a long time of experience.

**Methods:**

Information about the diseases and the ethnoveterinary practices performed was collected from a total of 90 herders and healers by interviews and participant observations.

**Results:**

The respondents classified the diseased in major and minor fractions. Clinical signs were given in detail. Mange followed by trypanosomosis and orf were considered the most prevalent diseases, and also caused the greatest economic losses. Orf was regarded the most complex disease. The season was considered to have great influence on the occurrence of the diseases. A variety of different treatments were described, such as medicinal plants, cauterization, odorant/fly repellents, pesticides, larvicides, *cold drink*, yogurt and supportive therapy (*hot food*, *hot drink*).

**Conclusions:**

There is paramount need to document and validate the indigenous knowledge about animal agriculture in general and ethnoveterinary practices in particular. This knowledge is rapidly disappearing and represents a cultural heritage as well as a valuable resource for attaining food security and sovereignty.

## Background

Camelids are important and precious to pastoral people, who rely on them in a variety of ways. Camels provide milk during conditions where other animals cannot, and they serve as draught animals in a complex mountainous ecology [1]. Camel pastoralists live in remote areas, constantly moving their herds. The camels are usually reared on isolated rangelands with poor infrastructure, where the livestock keepers rely on locally adapted breeds and make small ecological footprints [2]. Camelids as such cause less environmental harm compared to other livestock species [3].

The pastoralist way of living makes it very difficult to obtain veterinary extension services as in western countries. The allopathic veterinarians are usually trained in urban areas and inaccessible to the pastoral people, they are unfamiliar with the pastoralist traditions and terminology, and also have a lack of motivation [4,5]. The medical drugs used in allopathic veterinary medicine are expensive and not available in the desert [6]. Altogether this puts a pressure on the pastoralists to rely on themselves [7] and to base animal health care on ethnoveterinary medicine (EVM).

EVM has roots from time immemorial [8] but the scientific literature on the subject is new [9-12]. Since the 1970's the number of scientific papers, book chapters, etc have exploded and EVM now comprises a large body of written scientific and practical information [13]. Herbal medicine is an important part of EVM but not the only one, and the synonym "veterinary anthropology" indicates the complexity of EVM [9]. It is crucial in EVM to enhance the normal adaptive and defensive functions of the body because the causal agent may be impossible to eradicate or eliminate.

EVM is performed by traditional livestock healers [14]. Many herders and farmers treat their animals themselves, especially if the disease is well known [15]. Traditional livestock keepers have vast knowledge about their animals [2], and their EVM knowledge and skills are transmitted orally, from parents and grandparents [16]. This represents a constant development resulting in a mélange, which is not entirely indigenous but rather"folk" medicine [9]. However, the EVM knowledge is today rapidly lost, due to breakdown of traditional systems for knowledge transmission. An increasing integration of commercial drugs into EVM may also pose a threat, although EVM may well integrate new elements [16,4].

There is a wide variety of EVM treatment principles, according to the cause of disease, such as cauterization [17]; bleeding [18]; fuzzing, and minor surgeries; crushed leaves [19], tobacco leaves and fish waste [20], and oil for skin ailments; wooden splints for fractured limbs; meat and grains; grazing/browsing on certain plants; the use of different ashes including bone ashes, tree tar, wood oil, mineral springs, sea water, sulfur, motor oil, bone marrow oil, sour milk etc [21]. The treatments are based on the ideas that all living creatures should live in harmony with their environment and need a balance of hot and cold, work and play, wakefulness and sleep, etc [22]. The remedies differ between communities but many are routine treatments among several peoples [9]. The same remedy is often applied to both animals and humans [23]. The practices in an area are closely linked to the local flora and fauna [16].

Descriptive studies and clinical trials on EVM in Pakistan have been published in the international literature [24-28] but this is to our knowledge the first one from the Suleiman Mountainous Region. This region is a historic area in the Balochistan province, which is an important cradle of animal domestication and the habitat of many indigenous livestock breeds. Balochistan consists of 46% of the national land mass and less than 5% of the human population of Pakistan. The province is the home of many pastoral communities and moving herds and flocks of Afghan nomads.

The aim of this study was to obtain information about EVM from dromedary camel pastoralist herders and healers in the Suleiman Mountains.

## Methods

### Study area

**Figure 1:**
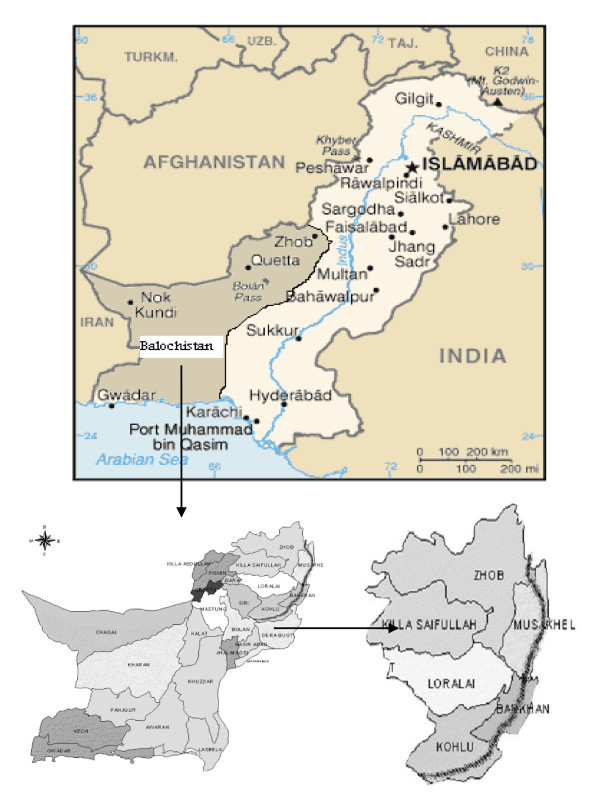
**Map of the study area in the Balochistan province**. The brush painted line indicates the Suleiman mountain series.

The Suleiman Mountainous Region (Figure 1), is arid to semi-arid land, receiving 200-500 mm precipitation bimodal. The region is situated from 29°37' to 31°70' at North latitudes and from 68°06' to 70°20' at East longitudes. The altitude is 600-1350 m above sea level. There are five seasons, i.e. spring, summer, autumn, winter and monsoon. The summer is warm (mean 21-32°C) and in the winter the temperature drops below 0°C. Rain (and occasionally snow) falls predominantly in the spring and the monsoon. Vegetation is sparse and consists of grasses, shrubs, bushes and trees. Woody vegetation of *Acacia modesta *is very common in the mountains, and other trees like Zizyphus, Olea and Pistachio are also prevalent. Bushes like *Caragana ambigua *and *Periploca aphylla *are found in the piedmont, while salt bushes like *Haloxyllon grifithi *and thorny vegetation of *Alhagi camelarum *are found in the plain areas. Grasses, mainly *Cynodon dactylon*, *Fraxinus xanthoxyloides *and *Stipa capillata *etc are also found.

The human population in the area comprises 0.5 million pastoral nomads, belonging to the Baloch and Pashtoon pastoral tribes. The economy is predominantly pastoral and the nomadic lifestyle has been practiced for centuries.

The pastoralists own herds of dromedary camels, cattle, sheep, goats and donkeys. About 46% of the dromedary camel population in Pakistan is found in Balochistan, out of which 30% in SMR (Table 1). The camel breeds are indigenous; two types of camel are found in the region, i.e. Kohi and Pahwali (Gaddai) [29]. The infrastructure of the area is very poor and camels are used for transportation in the remote areas. There are three major camel production systems in this region, i.e. nomadic, transhumant and sedentary. Camel shepherds may be hired for extensive grazing. The socio-economic importance of the camel is closely associated with the existing production systems, which are largely determined by climatic conditions, topography of the land, plant growth penology, water sources, etc [30]. There is no special arrangement of housing for the camels in this area. As the camels are always on move, they hardly spend more than one month at one place. They move according to the season and availability of the vegetation in the area.

**Table 1:** The camel population in different districts of the Suleiman Mountainous Region

*District*	*Male*	*Female*	*Total*
Barkhan	2 098	4 078	6 176
Killa Saifullah	6 369	4 558	10 927
Kohlu	24 796	23 647	48 443
Loralai	1 396	494	1 890
Musakhel	6 708	13 898	20 606
Zhob	2 343	844	3 187

			91 229

Source: [33]

### Respondents

A total of 90 dromedary camel herders and healers in Suleiman Mountainous Region were interviewed with a pre-tested questionnaire. Out of the 90 respondents, ten recognized experienced healers were selected for a detailed study of each disease. The healers were in average 47 years old and all were men except for one woman. They learnt EVM from their ancestors and by experience.

The interviews were performed during January 2008, and the ten experienced healers were accompanied in all five seasons to know all types of diseases and their time of prevalence. The first author accompanied and stayed with each respondent to observe and document the ethnoveterinary practices they conducted. The preliminary results were re-displayed to the respondents for feedback, and an in-depth group discussion was conducted for further validation and clarification of the results.

## Results and Findings

The major diseases were evaluated and ranked by the respondents (n = 90) according to their occurrence, their complexity and the resulting economic losses for each disease. The complexity was defined as the duration of the disease, the intricacy in treatment, the morbidity and mortality, and production losses for each disease [29]. The economic losses were defined as impaired production (work, draught, milk, growth etc), costs for drugs (costs for drugs, i.e. expenditure in the form of medicines, and duration of the diseases), enhanced cost for feeding (feeding charges, provision of supportive therapy and feeding at home), and caring/labor expenses (caring at home need special attention and care) [29].

### Diseases

**Figure 2:**
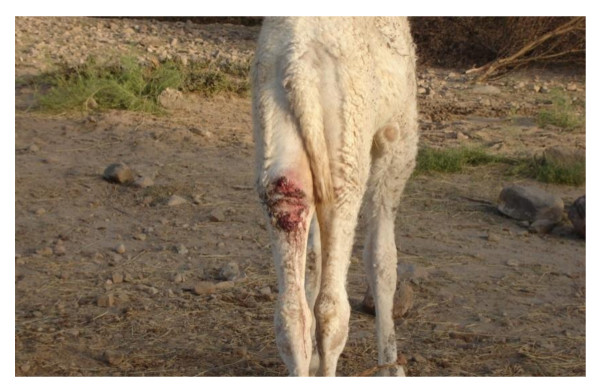
**Contagious skin necrosis (CSN) on the hindleg of a one-year-old camel calf**. Photo: Abdul Raziq.

**Figure 3:**
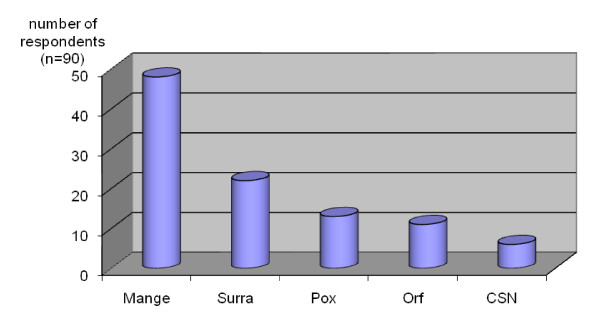
**Ranking of the occurrence of major diseases in dromedary camels as perceived by camel herders and healers**. (CSN = contagious skin necrosis).

The respondents stated that the disease panorama in camels is limited, i.e. camels do not suffer from as many different diseases as other livestock. The respondents have experienced mange, trypanosomosis, camel pox, orf and "Contagious skin necrosis"/"lymph node swelling" (dermatophilosis, corynebacteriosis, staphylococcosis) (Figure 2), which were regarded as major diseases, and ringworm, pneumonia, "hemorrhagic disease/haematuria", urinary obstruction, "febrile disease", mastitis, wry-neck syndrome and "poisonous disease" which were regarded as minor diseases. The occurrence of the major diseases is given in Figure 3. For further details on the description of etiology, clinical signs and diagnosis of the diseases see Appendix 1.

### Epidemiological aspects

**Figure 4:**
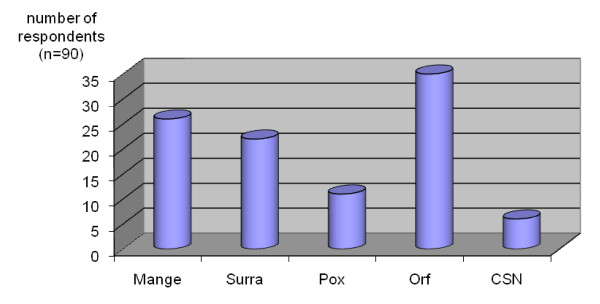
**Ranking of the complexity of major diseases in dromedary camels as perceived by camel herders and healers**. (CSN = contagious skin necrosis).

**Figure 5:**
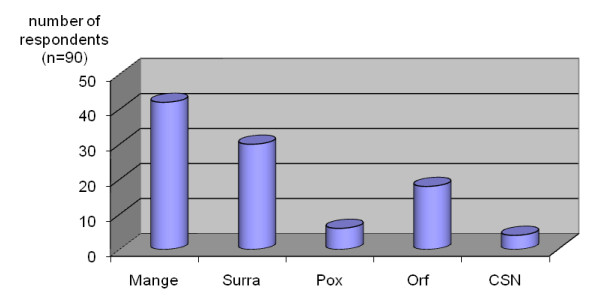
**Ranking of the economic losses for major diseases in dromedary camels as perceived by camel herders and healers**. (CSN = contagious skin necrosis).

The season of occurrence of different diseases is given in Table 2; the complexity of major diseases in Figure 4, and economic importance in Figure 5.

**Table 2:** Season of the camel diseases as given by the herders

*Disease*	*Season*	*Herders verification*
Mange	End of autumn	74%
Surra	End of monsoon	61%
Camel pox	End of spring	59%
Orf	End of spring	52%
Contagious skin necrosis (CSN)	Any time of the year	56%

### Treatments

For information about the terminology used by the respondents on ethnoveterinary treatments see Table 3.

**Table 3:** Treatments for camel diseases as described by herders and healers in the Suleiman Mountainous Region in Pakistan

Local name	English name	Description
Garam Khurak	Hot food	The herder's indigenous veterinary knowledge is mainly based on the hot and cold philosophy of food. The hot food comprises of soups made of cockerel meat, egg, pulses, cereals and chilies etc. Hot food is a composite of those nutrients which keeps the body active, energetic and enhance the activities inside the body. This soup is used for orf, pox and nodes swelling diseases.
		Supportive therapy (*hot food*), in which hot food (in most of the cases comprised of soups) is offered orally to keep the animal fit to deal with the disease condition.

Garam chishak	Hot drink	Hot drinks are used for the treatment of pulmonary problems and febrile conditions.
		A composite of the ingredients, which accelerate the biological activities in the animal body and enhance the secretion of the mouth, nose, and inside the body (GIT). Hot drink is considered as expectorant, laxative and diuretic. The solution is prepared by boiling black tea, ginger, and black pepper; sometimes chilies are also mixed. Black tea and spices mixture is also a hot food, which keeps the animal warm. This solution is mainly used in respiratory problems.

Kattan	Mud oil	The raw mud oil is collected from the natural spring in the famous mountain of Chakar Mangi, in Kohlu district. Kattan, which is thick in nature and blackish in color, is usually used by the Marri tribe camel herders for the treatment of mange in camel and goat.

Shlombey	Whey	*A *product of yogurt, when fat is removed by shaking and more water is added. The herder usually shakes the yogurt to remove fat and make shlombey in a sack (*Gharrak*) made of skin (usually from sheep). The solution remained behind is called Shlombey in Pashto.

Zarna	Wood oil	The Wood oil is extracted from the logs of Pinus Geranandiana tree. While burning the logs the released oil is collected in a pot.

Kirar	A type of tree	

Sorr Chishak	Cold drink	Cold drinks for trypanosomosis, food poisoning and poisonous plants treatment i.e. extract of *therkha *plant (*Artemisia maritima*)***, ***seed of *khamazura *(*Withania coagulans*) and sugar/gur juice.

Botai	Medicinal plants	Plants extract or crushed leaves or twigs, either applied directly on the affected area or administrated orally.

Dum	Hot branding	Hot iron brand or hot stone is applied either directly on the affected area or on the place considered being affective if branded. This treatment is done in most of the cases where the disease treatment is not easily recognized. This type of treatment is also called cauterization.

Boijan	Odorant	Odorant/fly repellents and larvicides etc, in which wood oil, used engine oil, kerosene oil, mud oil, smoke dust, ash and sometimes DDT or trichlorofons are used. This practice is mainly done in the treatment of skin ailments and wound management.

DDT/trich	DDT/trich	Odorant/pesticides and larvicides etc.

Thorkani	Ceiling smoke dust	Fly repellents and larvicides etc, healing effect

Thambako	Tobacco powder	Fly repellents and larvicides etc.

Ghee	Butter	Energetic; softens the area of the skin where ghee is applied

Gur	Sugar/gur	Energetic; cold effect

#### Mange

The treatment starts with washing, rubbing and cleaning of the skin with sand or other rough material followed by washing with laundry soap. The scabs and dirt are removed and the skin becomes clean and red. A mixture is made and applied on the affected areas. The mixture can be one or more of the following alternatives:

-Natural raw mud oil (*Kattan*) mixed 1:1 with *Taramira *(*Eruca sativa *seed) oil (Table 4).

**Table 4:** Ethnoveterinary plants used by dromedary camel healers in the Suleiman Mountainous Region in Pakistan

Botanical name	Local name	Part of plant used	Preparation, dose etc
*Eruca sativa* *Mill*. *[Brassicaceae]*	Taramira	Oil of the seed	The oil is applied on the affected area

*Daphne gnidium [Thymelaeaceae]*	Loghone	Leaves and twigs	The leaves and twigs are crushed, kept in a bowl over night in open, and pasted on the affected area

*Capparis aphylla Hayne ex Roth. [Capparaceae]*	Kirar	Leaves and twigs	Applied on the affected area

*Artemisia maritima L. ex Hook [Asteraceae]*	Therkha	Upper parts of the plant	500 g is boiled in one liter of water and given orally.

*Rhazya stricta Decne. [Apocyanaceae]*	Orgalama	Roots	Roots are burned and the ash is poured in the wound to kill maggots. The dose depends upon the depth of the wound

*Acacia modesta Wall*. *[Fabaceae]*		Spines	The spines are inserted in the wound

*Pinus gerardiana Wall. Ex D. Don [Pinaceae]*	Zarna	Wood oil	The logs of the tree are burned in a clavin-like muddy structure and the oil is collected and applied on the affected area. The dose depends on the size of the affected area.

*Whitania coagulans (Stocks) Dunal [Solanaceae]*	Khamazura	Fruits	The fruits (250 g) of the plants are dissolved in water and offered in the morning for trypanosomiasis.

-Wood oil (*Zarna*) mixed 1:1 with *Taramira *oil.

-Pulverized fresh chopped leaves and twigs of Loghone (*Daphne gnidium*) plant mixed with water or oil.

-Ash from Kirar (*Capparis aphylla*) wood soaked with *Taramira *oil and repeatedly applied until the lesion is dry and the skin becomes soft and smooth. Usually the animal is recovered within 1 month.

-Ceiling smoke dust soaked with *Taramira *oil.

-DDT or Trichlorofon powder added to used engine oil. Recovery time is one month.

-Tobacco powder dissolved in water. The application of tobacco powder and engine oil on cracked skin is very painful and sometimes fatal.

#### Trypanosomosis

The treatment strategy of trypanosomosis is based on (the thought of) neutralising the poison in the blood, awakening the camel's sleeping body and keeping the affected camel strong and fit. The poison in the blood is considered to be neutralised with the bitter taste of plants. To awake the sleeping body of the affected camel, branding at the base of the ear by hot red iron is practiced. Supportive therapy to stop the progressive emaciation and keep the affected camel as fit as possible to face the illness is also practiced. The astringent plant *Tharkha *(*Artimisia maritimae*) is thus recommended. The plant is crushed, boiled, kept in basin overnight and the extract is administrated orally as a *cold drink *once a day early in the morning. Three to four times treatment is believed to be effective. A soup of boiled, well thrived sheep meat can be given orally as supportive therapy.

#### Camel pox

Supportive treatment can be given to affected camel calves, usually a soup which provides liquid, energy and protein. The soup can consist of different ingredients which are boiled and thrived well, and a bowl (almost one liter) of soup is offered to the affected animal daily until recovery. The ingredients can be hen or cockerel (hen soup), head of sheep, goat or cattle (head soup), flour of Sorghum with spices and chilies (sorghum soup) or whole stomach compound of a small ruminant (stomach compound soup).

#### Orf

The strategy is based on specific treatment combined with supportive therapy. Specific treatment can consist of pouring warm, boiled water on the animal's head, or hot branding of the head. As pesticide and/or larvicide, application of DDT powder or Trichlorofon in kerosene oil on the lesions is used, or insertion by a smooth stick with ash from the burned root from the plant Orgalama (*(Rhazya stricta*). Some respondents complain/comment on that the specific treatment is not very effective against orf. Supportive therapy can consist of giving *hot food*.

#### "Contagious skin necrosis"/"lymph node swelling" (dermatophilosis, corynebacteriosis, staphylococcosis)

The affected animal is treated to enhance growth, maturation and rupturation of abscesses, and to stop access of flies to the wounds and discharge; the animal is also given supportive therapy. *Hot food *is considered important. Pulses and cereal or cockerel soup is offered to support animal health by additional energy and protein. Such nutrients are believed to increase the size of small abscesses and facilitate the rupturing of the mature ones. Spines of a tree (*Acacia modesta*) or needles can be used to puncture abscesses with delayed rupturing. Fly repellent like DDT or kerosene oil mixed with used engine oil are applied on wounds and ruptured abscesses.

#### Ringworm

Ringworm is considered to heal spontaneously but for treatment any oily material like *ghee *or diesel can be applied on the lesions.

#### Pneumonia

Generally no housing or shelter is provided to healthy animals in the area, but animals suffering from pneumonia are kept in a covered area, protected from cold weather by a carpet, rug, or blanket. The animal is given *hot food*, and *hot drink*, i.e. a boiled mixture of black tea and spices. Dyspnea due to obstruction of the nasal airways is cured by insertion of a stick in the nose to facilitate breathing. Branding with hot stones on both sides of the thorax is performed.

#### "Haemorrhagic disease/haematuria"

The camels are treated by giving orally the ash from animal bones (any species) and/or with branding by red-hot iron or hot stone at both flanks.

#### Urinary obstruction

The treatment aims at to facilitate urination, i.e. enhanced frequency and increased amount of urine. Different liquids are given orally; a solution of crushed alum (50-100 g, preferably red) dissolved in water, *hot drink *(black tea) or an extract of boiled wheat straw. Another treatment that is practiced is to keep the camel for an hour in sitting posture with the whole body but the head under water.

#### "Febrile disease"

**Figure 6:**
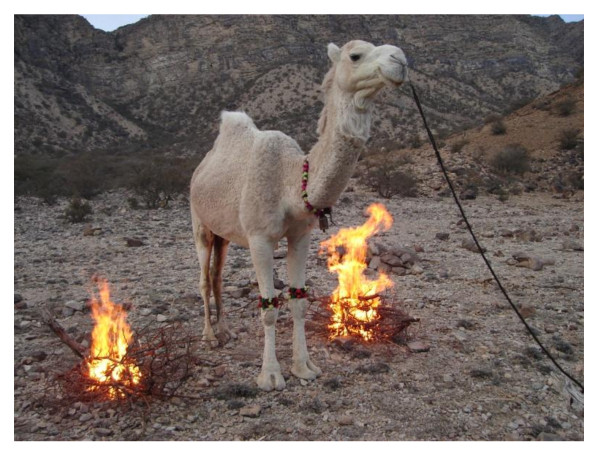
**Camel under treatment for febrile disease (Oshmak)**. Photo: Abdul Raziq.

There is no specific treatment, but to provide heating. The diseased camel is placed standing between two flames for one hour (Figure 6). Sometimes a Muslim priest prays for the camel. As supportive treatment, yogurt or *shlombey *is given orally. One liter of yogurt or two liter of *shlombey *with salt added is offered 3-4 times daily to the affected animal, while drenching it with sugar solution. In prolonged cases, sheep meat soup is offered.

#### Mastitis

The treatment of mastitis is oil massage of the udder and to provide heating by application of a hot lump of mud. Occasionally the mammary vein is bled.

#### Wry-neck syndrome

The only treatment is considered to be giving ash orally plus some supportive therapy.

#### "Poisonous disease"

The camel is treated to reverse the indigestion and the effect of the poison. Two liters of salt added *Shlombey *or one liter of *hot drink *is given orally twice with four hours between.

## Discussion and Conclusions

The herders and healers in the Suleiman Mountainous Region have deep knowledge and vast experience of camel husbandry. They regard camels as hardy and healthy animals with only a limited disease panorama compared to other livestock animal species. They are familiar with the clinical signs of camel diseases and they can differentiate between many diseases. Living in remote areas, they have developed their own way of treating diseases in camels. A variety of methods of treatment was documented in this study, many of them much alike those reported from other camel habitats worldwide [10]. Some methods are very specific, e.g. to use *Acacia *spines to puncture abscesses, while other are more general, e.g. supportive treatment to promote health and make the animal more fit to combat the disease. The respondents regard ethnoveterinary practices as reliable, painless, cheap, readily available and easily applicable.

From a veterinary point of view, EVM has its strengths and weaknesses. Not all ethnoveterinary practices provide effective or ideal solutions to animal health problems - no more than does allopathic veterinary medicine [10]. However, allopathic veterinary medicine would benefit from taking up the holistic and sustainable view in EVM [22], and also adopt a respectful attitude towards the fact that herders have their own considerations and social believes e.g. about the causes of diseases [9], [31]. Many EVM practices do work and make sound veterinary sense, and many modern drugs have their origin in EVM [10,32].

EVM is rapidly disappearing in the Suleiman Mountainous Region and other livestock keeping areas because of social, economical, and political reasons. Important factors in Balochistan are e.g. speedy urbanization, increasing sedentarization, changing livestock systems from subsistence (extensive) to commercial (intensive), economic forces and political backing for crossbreeding with exotic breeds, war and conflicts, increasing competition for natural resources, environmental degradation and global warming.

There is paramount need to document and validate ethnoveterinary practices and other indigenous knowledge in Balochistan, which is an important region because of the large number of livestock animals, dromedary camels in particular, and because EVM and other Indigenous knowledge is still used in practice. The launching of regional and international projects like e.g. the RUBIA project in the Mediterranean countries would help to conserve and validate indigenous and ethnoveterinary knowledge and thus contribute to global food security and sovereignty.

## Competing interests

The authors declare that they have no competing interests.

## Authors' contributions

AR was responsible for conception and design, carried out the questionnaire and observational studies and drafted the manuscript. KV helped to draft the manuscript. MY provided guidance and participated in discussions. All authors read and approved the final manuscript.

## Appendix

### Appendix 1: Descriptions of the knowledge on diseases, given by dromedary camel herders in the Suleiman mountainous region

The local names of the diseases are written in bracket, where the first name is in Balochi and the second name is in Pashto

**Mange (Gerr, Poon) **is a well-recognized and common disease in the area and one of the major dromedary camel illnesses. The cold and rainy weather and scarcity of vegetation in the early spring make animals weak, hence more prone to this disease. Mange is therefore more prevalent in the cold and rainy season, when the animals are already weak. Animals affected by trypanosomosis are also prone to mange; because of its weight-loss nature. Mange affects the fertility and lesser number of calves was produced in the herds where the mange intensity was high. The area has cool winter and rainy spring, which enhances mange. The respondents revealed that the disease is contagious, widespread, causes poor growth and production, affects the draught ability, and even causes the death of the animal in severe cases. The healers believe that the disease is highly contagious and zoonotic in nature and transfer even from the rats. During the last drought period in the region (1994-2004) many animals died due to mange.

There are two types of mange, i.e. white and black. The white mange is milder than the black one and covers a certain area. Animals are itching the body against hard objects, the skin becomes thick and bald and whitish scabs appear on it. In black mange the baldness covers major parts of the body, the skin becomes red-blackish and muddy, cracks appear and start bleeding, and the animal become emaciated. The cracks usually appear on the neck area, which bleeds and invite flies, making the animal restless. This type of mange is very hazardous, complex and causes fatalities also.

The mange-affected animals are usually rejected in the camel markets because of the low credibility for work. The traders know the mange-affected animal very quickly by rubbing the skin of the lower neck and judging the thickness of the skin. The affected animals have thick skin because of mange.

**Trypanosomosis/surra (Sokerr, Machwahali) **is not as common as mange but badly affects the draughtability and fertility of the animals. Loss of appetite, progressive emaciation, hump disappearance, distinct urine smell, watery and pale eyes, intermittent fever, rough body coat and sitting while facing the sunshine are the main symptoms. Trypanosomosis is economically important and affects the animal health and production adversely. Outbreaks occur after the rainy season, when there are plenty of flies. The pastorals are well familiar with trypanosomosis and believe that a fly is the carrier of this ailment.

**Pox (Groopak, Zazi) **is a highly contagious disease, which usually affects the calves, and outburst in the wet season particularly in spring. The disease is also known in the region as the spring's disease. Pox predisposes the animals to other diseases like trypanosomosis and orf. The mortality is very low, only continuous off feeding and secondary infection could cause death. The animal becomes dull and depressed in the onset of the disease, gets fever, and lesions appear on the hairless parts of the skin. The animal goes off feed, unable to eat due to lesions in the mouth and the lips. The disease could be differentiated from orf, because in orf the lesions only appear on the mouth, nose and eyes. Pox occurs once in life and will never repeat again and the phenomenon is indigenously called "pokh".

**Orf (Duph Pagh, Serpazi) **is also "pokh" in nature, i.e. occurs once in the life. Orf occurs before the permanent teeth appear. The disease is contagious coupled with fever and depression. Nodules develop on the lips and changes into blisters. In advanced stages blisters are formed inside the mouth and nose. Swelling of the face and the head is the third and the advanced stage of orf. If not treated properly, the animal becomes blind and unable to eat.

**Contageous Skin Necrosis (Rindek/Jooling, Daney) **affects all ages of animals but young animals are more prone. The respondents consider that the disease is good for the future health of animals, because it drains the unidentified disease factors with the purulent fluid. The herders, therefore, offer *hot food*, to keep the occurring wound pustules. When the pus is later discharged then the animal recovers.

The sores usually appear on the soft areas of the body i.e. neck, shoulders and thighs. The lymph nodes also swell with the disease. Fever, dry and hard feces, off feeding and emaciation were also observed. Gradually the abscess increases in size and ultimately reaches the size of an apple. The large sized abscess bursts itself, but is sometimes punctured by injecting of a large-sized needle. Small-sized abscesses evaporate themselves, but take more time than pustulated abscess. If the abscess could not grow well it would affect the inside of the body and the disease will go in hidden form, which will adversely affect the animals' health. A yellowish viscous fluid discharges when bursts and become pustulated for some days, in this period fly repulsion is the important part of the disease management.

**Ringworm (Barri, Spooni) **is also called "sehsali", which means "the disease of three years"; this disease usually comes in calves up to the age of three years, and is widespread in the herd. White patches starts from the tip of the hump and progress down to the belly. Hair-loss in patches and white flour scabs appear on the affected area. The disease usually come in the early spring and can easily be confused with mange, but this disease heals itself.

**Pneumonia (Kalokh, Tookha) i**s not very common but adversely affects the animals' health, especially the working animal. It is characterized by nasal discharge, coughing and difficulty of breathing. Sometimes the nose is obstructed due to the thick nasal fluid-like cartilage and the animal breathe through mouth. The respondents believe that pneumonia is caused by cold and dusty winds in the autumn.

**Hemorrhagic diseases or heamaturia (Lalmis, Sary mithiyazi) **is not common in the area and only occurs in mature females. The disease results in weakness and emaciation of the animal, and the urine becomes blood-stained. The herders could not explain the disease well. The respondents have only a vague explanation of the cause of this disease, some kind of deficiency or kidney problem are suggested. The clinical signs are described as haematuria, depression and emaciation.

**Urine obstructions (Misband, Mithyaziband) **are mainly noticed in male draught animal. The causes of the disease are heavy workload, long traveling, dirty water, severe cold and poisonous leaves of wild olive (*Olea officinalis*). The herders believe that the wild olive leaves become poisonous in the fall season. The animal goes off feed and water, and loses the capability to work. The animal continuously tries to urinate but does not succeed, only sometimes some drops of urine come out.

**Febrile Disease (Oshmak, Shumak) **is not common but sometimes very fatal for the camel. The cause of "febrile disease" is not fully clear to the respondents, although they indicate that an insect present in the leaves of trees may be a causative agent. Some respondents believe that the causes are the evil eye and dirty air. The respondents describe the clinical symptoms as vomiting, weakness, emaciation and fever. Anorexia and dislike and avoidance of sunshine are other signs. Acute death may occur and some of the respondents consider anthrax as the differential diagnosis.

**Poisonous disease (Marzal, larrama) **mostly occurs after the monsoon season, in the start of the autumn. The respondents consider the cause of the disease to be ingestion of a poisonous insect hiding in the leaves of the Karkana (*Zizyphus nummolaria*) and *ber (Ziz mauritiana) *trees. The clinical signs are severe indigestion, anorexia, vomiting and fever. Sometimes the outcome is fatal.

### Poisonous plants

The respondents consider three plants as poisonous for the camel. The local and botanical names are Kaneer/Genderi (*Nierum odorum*), Leghunae (*Daphne oleoides*), and Orgalama (*Rhazya stricta*).

### Wry-neck syndrome

Wry-neck syndrome is not familiar to the respondents. Some of them consider poisonous plants and some deficiency to be the cause of wry-neck syndrome. The clinical signs are described as the neck of the camel becoming tight and the animal is unable to drink water and intake food properly.

### Mastitis

Mastitis is very rare in the dromedary camel because of the extensive production system and living in movement and on the clean lands. Sometimes problems with mastitis occur, especially in high producing camels.

## Supplementary Material

Additional file 1 **Names of the respondents**. The file contains the names of the respondents (herders and healers) who provided the information reported in this study on ethnoveterinary practices in the Suleiman Mountainous Region in Pakistan. Click here for file
