# Supplementary material for: Ethnoveterinary treatments by dromedary camel herders in the Suleiman Mountainous Region in Pakistan: an observation and questionnaire study
Source: J Ethnobiol Ethnomed. 2010 Jun 21;6:16. doi: 10.1186/1746-4269-6-16 (PMC3224957; doi:10.1186/1746-4269-6-16)
Supplement: Additional file 1 — Names of the respondents. The file contains the names of the respondents (herders and healers) who provided the information reported in this study on ethnoveterinary practices in the Suleiman Mountainous Region in Pakistan. [file 1746-4269-6-16-S1.PDF]

Additional file. Names of the respondents of the Suleiman Mountainous Region in Pakistan

No	Name of area	No of camels
1	Abdullah	7
2	AKhtar shah	12
3	Al Lehma	60
4	Allah Bux	10
5	Alo Gul	8
6	Andar	51
7	Asal Khan	5
8	Babi	9
9	Baran	12
10	Baras	9
11	Batgai	11
12	Bazo	40
13	Dino	200
14	Dostha Gul	7
15	Dotani	9
16	Esa Jath	5
17	Galote	8
18	Ghairat	5
19	Golai	41
20	Gul	15
21	Gulbaz	22
22	H. Khanan	34
23	H. Haider	7
24	H. M. Jan	15
25	H. Nazar Khan	7
26	Haji Dadan	33
27	Hakeem	41
28	Hanif	28
29	Hassan	56
30	Hothan	21
31	Ibrahim	5
32	Jamal	5
33	Jan Muhammad	6
34	Janan	7
35	Jano	62
36	Kabul Khan	12
37	Kakar	16
38	Kala Khan	17
39	Karim	19
40	Khair Mah	30
41	Khair Muhammad	7
42	Khairo	6
43	Khial	8
44	Khiro	69
45	Lalai	5
46	Lalak	4
47	Lateef	21
48	Loghuni	38

49	Louon	34
50	Mago	41
51	Malak	23
52	Mamo Jan	5
53	Marwat	7
54	Meero	8
55	Mehma Khan	36
56	Mira	40
57	Mughal	15
58	Muhammad Din	12
59	Murad Ali Rind	13
60	Musa Gul	17
61	Nasar	19
62	Noora	8
63	Pato Khan	9
64	Qaim Khan	5
65	Qandhari	7
66	Qathal Khan	17
67	Razo	6
68	Sadiq	9
69	Sakar	27
70	Sakhi	35
71	Sakul	81
72	Salah	50
73	Santhul	7
74	Shamar	8
75	Sheikh	5
76	Shero	90
77	Shinkai	70
78	Sohrab	33
79	Sotie	24
80	Suleiman	41
81	Syed	6
82	Thora Jan	13
83	Umar	8
84	Umerzai	57
85	Wadera Rahzan	17
86	Yaqoob	8
87	Zadran	6
88	Zar Gul	9
89	Ziarat	7
90	Zulmi	11
